# When style meets strategy: testing the moderating role of regulation strategies in the association between emotional style and self-conscious emotions

**DOI:** 10.3389/fpsyg.2026.1913847

**Published:** 2026-09-09

**Authors:** Corinna Elisabeth Burgstedt, Daniela Hosser

**Affiliations:** Institute of Psychology, Technische Universität Braunschweig, Braunschweig, Germany

**Keywords:** emotion regulation, emotional style, shame, guilt, interaction effects, moderation, moral emotions

## Abstract

**Introduction:**

Shame- and guilt-proneness represent relatively stable tendencies to experience self-conscious emotions following perceived transgressions. Shame-proneness has traditionally been linked to negative self-evaluation, whereas guilt-proneness has been associated with responsibility-taking and repair. However, both tendencies are increasingly considered in relation to broader emotional functioning and regulatory processes.

**Methods:**

This cross-sectional study investigated whether the emotional-style dimensions outlook (sustained positive affect) and resilience (recovery from negative emotional states) are associated with shame- and guilt-proneness, and whether cognitive reappraisal and suppression moderate these associations. Participants (*N* = 331 adults) completed an online survey including the Questionnaire of Emotional Style (FEMOS), the Test of Self-Conscious Affect (TOSCA-SP), and the Emotion Regulation Questionnaire (ERQ).

**Results:**

Structural path analyses showed consistent negative associations of positive outlook and resilience with shame-proneness, whereas suppression was positively associated with shame-proneness. Outlook was also positively associated with guilt-proneness in the model including suppression but not in the model including cognitive reappraisal. Overall, shame-proneness appeared to be more consistently associated with broader emotional functioning, including individual differences in maintaining positive affect, recovering from negative emotional states, and habitual suppression of emotional expression. In contrast, guilt-proneness showed a more selective pattern of associations involving positive outlook and cognitive reappraisal, potentially reflecting a more constructive orientation toward processing and responding to perceived transgressions. However, none of the tested interaction effects reached significance, indicating that habitual emotion regulation strategies did not significantly moderate the associations between emotional style and shame- or guilt-proneness.

**Discussion:**

These findings suggest that different aspects of emotional functioning and regulation contribute to individual differences in self-conscious emotional experiences, while their interplay may be more complex than assumed.

## 1 Introduction

Emotion regulation is defined as the capacity to monitor, evaluate, and modulate one’s emotional responses in a manner that aligns with the demands of the environment and situational goals. It is a central factor for mental health, wellbeing, and successful adjustment to environment (e.g., Aldao et al., 2010; Erisman and Roemer, 2010; Gratz and Roemer, 2004; Hu et al., 2014; Kraiss et al., 2020). Consequently, emotion regulation is a frequent target in psychotherapy with patients with mental disorders (Iwakabe et al., 2023).

Two emotions that are particularly relevant for social adaptation are shame and guilt, which are commonly referred to as self-conscious emotions (Barrett, 1995; Roos, 2000; Tracy et al., 2007). Both can be elicited by violations of internalized conventions, norms, or laws (Roos, 2000). Consequently, they are also of considerable relevance to social and antisocial behavior. This is particularly evident in the context of prevention, intervention, and rehabilitation, including early risk identification and education aimed at reducing antisocial conduct, programs targeting maladaptive cognitions and affect regulation to reduce problematic behavior, and treatment-based support and reintegration following antisocial or criminal behavior aimed at sustaining behavior change (Ewald, 2018; Hosser et al., 2008; Spruit et al., 2016; Tangney et al., 2007a, 2011a, 2011b, 2014; Tibbetts, 2003).

A promising framework for gaining a deeper understanding of emotion regulation processes is offered by the concept of emotional style (Davidson and Begley, 2012; Kesebir et al., 2019). It corresponds to the scientific understanding of temperament (Burgstedt et al., 2025), as it describes dispositional tendencies toward certain emotional and behavioral responses that are already observable in childhood and adolescence. According to the conceptualization of emotional style, six dimensions determine the occurrence, quality, and intensity of emotions and how these change over time: outlook, resilience, self-awareness, social intuition, sensitivity to context, and attention (Davidson and Begley, 2012). They can be regarded as relatively stable and fundamental determinants for processes of emotion genesis and regulation. At the same time, due to high neural plasticity, they are likely malleable and amenable to change. Modifying these relatively stable determinants may translate into broad downstream changes across emotion genesis and regulation. Therefore, emotional style-informed approaches may offer an advantage over conventional emotion regulation trainings that primarily target individual, learnable skills (e.g., Berking, 2010; Berking and Whitley, 2014; Heinrichs et al., 2017), potentially serving as a powerful leverage point for interventions with large, broad, and lasting effects. In particular, the dimensions outlook and resilience have been associated with various indicators of psychological and physical wellbeing, such as life orientation, difficulties in emotion regulation, positive and negative affect, depression and anxiety, stress, physical symptoms, subjective vitality, satisfaction with life, and quality of life, as well as self-perceived success in wellbeing-relevant domains (e.g., relationships, self-esteem, and purpose in life) (Burgstedt et al., 2025; Gasiorowska et al., 2023; Jekauc et al., 2021; Kesebir et al., 2019; Malandrone et al., 2022; Nazari and Griffiths, 2022). Outlook and resilience may also be relevant for self-conscious emotions because they capture individual differences in maintaining positive affect and recovering from emotionally challenging experiences. These processes may shape how individuals process and regulate self-conscious emotions elicited by experiences that challenge their self-concept, moral standards, or social bonds. A more positive emotional baseline (positive outlook) and faster recovery from negative affective states (high resilience) may support a more stable positive self-concept and a more optimistic, action-oriented response after a moral failure, often associated with guilt-proneness (Tracy and Robins, 2004, 2006), while reducing negative rumination and self-devaluation, often associated with shame-proneness (Tracy and Robins, 2004, 2006). Although these emotional style dimensions do not capture the moral appraisal processes underlying shame and guilt, they may contribute to dispositional differences in how these self-conscious emotions are experienced and regulated.

Compared with emotional style dimensions, emotion regulation strategies, such as cognitive reappraisal and suppression, are strongly shaped by socialization processes (McRae and Gross, 2020; Wright et al., 2025). Emotion regulation strategies refer to distinct cognitive and behavioral patterns employed to modify emotional responses. They can be acquired throughout development and across the lifespan, and are often explicitly taught in psychotherapeutic interventions and experimental training paradigms. Cognitive reappraisal and suppression are also assumed to influence the experience of shame and guilt (Xu, 2024). Cognitive reappraisal involves changing the interpretation or meaning of emotional events (Gross and John, 2003). A positive reinterpretation of the event and one’s contribution may be associated with long-term reductions in dysfunctional negative emotions and with enhanced positive affective functioning following own moral transgressions, thereby promoting adaptive guilt-proneness and reducing shame-proneness. In contrast, suppression refers to inhibiting emotional expression after the emotion has already been generated (Gross and John, 2003) and may be linked to increased shame-proneness, as the underlying negative affect elicited by the event is not altered in the long term. Consequently, suppression may also make adaptive forms of guilt less likely.

Importantly, temperament theories emphasize the interplay between emotional reactivity tendencies and regulatory processes, suggesting that the way individuals regulate their emotional responses may influence how these tendencies relate to psychological outcomes (Rothbart and Bates, 2006; Eisenberg et al., 2010). While these theories conceptualize regulatory capacities as a more general aspect of temperament, individual emotion regulation strategies can be understood as specific learned skills for managing emotional responses (Wright et al., 2025). Previous research has already shown that the consequences of dispositional emotional reactivity may vary depending on how individuals habitually regulate their emotional responses (Shapero et al., 2016). With reference to the emotional style dimensions of outlook and resilience, vulnerability to maladaptive shame-related processes associated with low outlook and resilience may be further intensified when emotions are habitually suppressed. Conversely, cognitive reappraisal may buffer this vulnerability by fostering more adaptive interpretations, thereby reducing shame-related processes and promoting more adaptive guilt-related responses.

Accordingly, the interplay between a person’s fundamental emotional style and the use of specific emotion regulation strategies warrants closer examination. To date, it remains unclear to what extent the influence of temperament on the proneness to self-conscious emotions can be amplified or attenuated by such strategies.

The present study addresses this gap by examining whether outlook and resilience, as dimensions of emotional style, predict shame- and guilt-proneness. Furthermore, it investigates whether these associations are moderated by the emotion regulation strategies of cognitive reappraisal and suppression.

## 2 Theoretical background

### 2.1 Emotional style: outlook and resilience

The concept of emotional style was developed by Davidson, who proposed that individual differences in emotional functioning are associated with relatively stable patterns of neural activity involved in emotion regulation and behavior (Davidson and Begley, 2012). This concept shares important conceptual similarities with developmental theories of temperament, particularly Rothbart’s model of temperament (e.g., Rothbart, 2007; Rothbart and Bates, 2006; Rothbart and Derryberry, 1981). In this framework, temperament refers to relatively stable individual differences in emotional reactivity and self-regulation that emerge early in development and contribute to later patterns of personality and psychological functioning (Rothbart, 2007). These differences encompass variations in emotional, motor, and attentional reactivity, as well as in regulatory capacities, and are understood to reflect underlying biological systems while remaining responsive to environmental experiences and developmental influences (Buss and Plomin, 1984; Pedlow et al., 1993; Rothbart et al., 2000). Thus, although temperament shows substantial continuity across the lifespan, it is not viewed as fixed but as a dynamic system that is continuously shaped by interactions between individual predispositions and environmental contexts.

Davidson identified six dimensions, each of which corresponds to specific activation in different brain regions (for an overview of the various neural activity patterns, see Davidson and Begley (2012) and Kesebir et al. (2019)). With regard to numerous outcomes—including wellbeing, life satisfaction, positive and negative affect, and depression—two dimensions have consistently emerged as particularly relevant: outlook and resilience (Burgstedt et al., 2025; Gasiorowska et al., 2023; Jekauc et al., 2021; Kesebir et al., 2019; Malandrone et al., 2022; Nazari and Griffiths, 2022).

Outlook describes the ability to sustain positive emotions over time (Davidson and Begley, 2012; Kesebir et al., 2019). When these emotions are consistently maintained rather than experienced momentarily, they gradually consolidate into a stable, optimistic outlook on life and its future possibilities. This basic disposition is defined at the neural level by an individual’s capacity to sustain activity in brain regions associated with positive affect and reward, particularly the nucleus accumbens (Heller et al., 2009).

Resilience refers to the capacity to recover quickly from stress (Davidson and Begley, 2012; Kesebir et al., 2019). Individuals who quickly regain equilibrium after minor everyday stressors also tend to rebound more rapidly in the face of significant life adversities. High levels of resilience are associated with more robust functional coupling between the prefrontal cortex and the amygdala, reflecting enhanced regulatory control over emotional responses (Davidson and McEwen, 2012; Kim and Whalen, 2009).

Outlook and resilience may be particularly related to emotional recovery processes and the interpretation of emotionally challenging situations because they capture two complementary aspects of how people deal with aversive input over time: resilience refers explicitly to how quickly negative affect is recovered after emotionally challenging or aversive experiences, whereas outlook reflects the degree to which individuals maintain a positive emotional baseline and expectations even in the face of setbacks (Davidson and Begley, 2012; Kesebir et al., 2019). In contrast, other emotional style dimensions, such as social intuition, self-awareness, sensitivity to context, and attention, primarily concern how individuals detect and process cues, allocate attention, and monitor internal and external states, but they do not capture recovery-related dynamics and the maintenance of positive affect in the same direct way. Accordingly, when individuals experience moral transgressions as particularly self-relevant setbacks, lower positive outlook and lower resilience are expected to contribute to more persistent negative emotional tone and slower affective recovery and, whereas higher levels of positive outlook and resilience should support more positive interpretation of the situation and faster emotional recovery.

### 2.2 Emotion regulation strategies: cognitive reappraisal and suppression

Factors that may strengthen or weaken the effects of temperament-based emotional dispositions include emotion regulation strategies. It is assumed that emotion regulation involves not only different types of strategies, which may be applied consciously or operate automatically (Gross, 2015), but also habitual preferences for the use of specific strategies (Abler and Kessler, 2009; Gross and John, 2003). Importantly, emotion regulation strategies are generally regarded as being more strongly influenced by socialization and learning processes (McRae and Gross, 2020; Wright et al., 2025). Two strategies that are frequently used are cognitive reappraisal and suppression (McRae and Gross, 2020). There is disagreement as to whether the former can be considered an anticipatory and the latter a reactive strategy (Abler and Kessler, 2009) or whether such a categorization and classification should be considered outdated, as both strategies can be used at different times in the development of emotions (Heber et al., 2014). Empirical findings show that within Western populations, cognitive reappraisal is often superior to suppression in terms of its functionality (Gross and John, 2003; Heber et al., 2014), even if both strategies could be used successfully in subjective perception (Gross and John, 2003).

The aim of numerous interventions in a therapeutic context is to reduce so-called maladaptive emotion regulation strategies in favor of adaptive strategies (McRae and Gross, 2020). Here, too, cognitive reappraisal in the sense of a positive reinterpretation is seen as an opportunity to develop a positive self-view (Lammers, 2016). Cognitive reappraisal is therefore an adaptive strategy (McRae and Gross, 2020) that can reduce the experience and expression of negative emotions (Abler and Kessler, 2009; Gross, 2002) and at the same time increase the experience and expression of positive emotions (Gross, 2002). Cognitive reappraisal can be conducive to coping with challenging situations and is also positively associated with psychological wellbeing and mental health (Gross and John, 2003; Hu et al., 2014) and negatively associated with psychopathological symptoms (Aldao et al., 2010; Cludius et al., 2020).

In contrast, suppression is considered maladaptive, also because an increased use of this strategy can be observed in patients with affective disorders (Aldao et al., 2010). At the same time, it is negatively associated with psychological wellbeing and positively associated with psychopathological symptoms (Cameron and Overall, 2018; Hu et al., 2014). It is assumed that, although a decrease in the subjective perception and visibility of emotions is possible in the short term by suppressing the expression of emotions, a medium and long-term reduction in physical and disruptive feelings is unlikely (Bock, 2013). There are even findings that suggest a strengthening of (unwanted) emotions through suppression (Berking, 2010).

### 2.3 Self-conscious emotions: shame and guilt

Shame and guilt belong to the family of self-conscious or moral emotions that arise when individuals evaluate themselves in relation to internalized social norms, values, and moral standards (Roos, 2000; Tangney et al., 2007a, 2007b; Tracy et al., 2007). The distinction between shame and guilt proposed by Lewis (1971) and further elaborated by Tangney and colleagues (e.g., Tangney et al., 2007a, 2007b; Tangney and Dearing, 2002) has been highly influential in explaining emotional responses to perceived failures or moral transgressions. Within this framework, shame is characterized by negative evaluations of the global self (“I am a bad person”), whereas guilt primarily involves negative evaluations of a specific behavior (“I did something bad”) (Tangney and Dearing, 2002). Accordingly, shame has been associated with global, stable, and uncontrollable self-attributions, whereas guilt has been linked to specific, unstable, and controllable appraisals of one’s behavior (Tracy and Robins, 2004).

More recent conceptual approaches have extended this distinction by emphasizing that shame and guilt are not entirely discrete emotional categories but overlapping, context-dependent experiences shaped by how individuals interpret their actions, themselves, and their relationships with others (Tracy et al., 2007; Singh and Bhushan, 2025). Although shame is typically characterized by global evaluations of the self and guilt by evaluations of specific behaviors, contemporary perspectives highlight that the emotional and behavioral consequences of both emotions depend on the broader context in which these appraisals occur (Tracy and Robins, 2006; Gilbert, 2007). Consistent with recent conceptualizations, guilt is no longer regarded as uniformly adaptive. Rather, constructive forms of guilt characterized by responsibility-taking and reparative motivation can be distinguished from maladaptive forms involving excessive self-blame and persistent rumination (Tilghman-Osborne et al., 2010; Abraham and Padmakumari, 2025).

Contemporary conceptualizations of guilt have distinguished between intrapsychic, interpersonal, and integrative perspectives (Abraham and Padmakumari, 2025; Carnì et al., 2013). The intrapsychic tradition, originating from psychodynamic theories, conceptualizes guilt as an internal experience arising from conflicts between personal impulses and internalized moral standards, with an emphasis on self-directed distress, punishment, and the regulation of internal states (Freud, 2005; Mosher, 1965; Rank, 1999). In contrast, interpersonal approaches conceptualize guilt as a socially embedded moral emotion that emerges from perceived harm to others or failures to meet interpersonal responsibilities, thereby promoting empathy, reparative behavior, and the maintenance of social relationships (Baumeister et al., 1994; Baumeister et al., 1995; Kubany and Watson, 2003; Tangney et al., 2007b). Integrative perspectives seek to reconcile these approaches by proposing that guilt may arise from distinct but related moral concerns, including altruistic guilt resulting from harm caused to others and deontological guilt resulting from violations of internalized moral principles (Mancini and Gangemi, 2021).

The present study acknowledges that guilt reflects both intrapsychic and interpersonal processes while focusing on a specific level of analysis. Using the gold standard self-report measure of shame and guilt, shame and guilt are operationalized as individual tendencies to experience these emotions in response to hypothetical interpersonal and moral transgressions (Ferguson et al., 2007; Tangney et al., 1989). Although these scenarios involve interpersonal contexts, the operationalization focuses on relatively stable individual differences in emotional responding rather than on the dynamic interpersonal processes through which shame and guilt unfold. Moreover, this operationalization primarily emphasizes maladaptive aspects of shame, such as global negative self-evaluation, and relatively adaptive aspects of guilt, including responsibility-taking and reparative action tendencies. Consequently, the present findings should be interpreted with reference to this specific operationalization as shame- and guilt-proneness rather than as reflecting the full phenomenology of shame and guilt.

## 3 The present study

Emotional style reflects relatively stable differences in emotional reactivity, regulation, and recovery processes (Davidson and Begley, 2012; Kesebir et al., 2019). These individual differences may also shape the persistence of emotional responses and recovery following moral violations or perceived failures. Existing evidence suggests associations between broader dispositional characteristics and shame- and guilt-related tendencies (Erden and Akbağ, 2015; Basu et al., 2025; Mu et al., 2026). However, previous studies have focused primarily on personality traits, such as the Big Five (Erden and Akbağ, 2015; Basu et al., 2025; Mu et al., 2026), or on externalizing and internalizing psychopathology (Mu et al., 2026), rather than on temperament-related dimensions.

The present study addresses this gap by examining whether emotional style dimensions are associated with shame- and guilt-proneness. Importantly, emotional style is not assumed to determine whether a given situation elicits shame or guilt, as these emotions are also shaped by situational and moral factors, as well as appraisal processes involving self-evaluation, responsibility, and perceived wrongdoing (Tracy and Robins, 2006; Tracy et al., 2007). Rather, emotional style may shape how individuals process and regulate these self-conscious emotions, contributing to variability in their habitual experience across situations. Among the emotional style dimensions, outlook and resilience may be particularly relevant because they reflect individual differences in sustaining positive affect even after setbacks and recovering from aversive experiences (Davidson and Begley, 2012; Davidson and McEwen, 2012; Heller et al., 2009; Kesebir et al., 2019; Kim and Whalen, 2009). Lower positive outlook or resilience may be associated with greater vulnerability to prolonged negative affect following perceived transgressions. Based on the present operationalization, which primarily captures global negative self-evaluation for shame-proneness and responsibility-taking with reparative action tendencies for guilt-proneness, a positive outlook and resilience were expected to be negatively associated with shame and positively associated with guilt.

Emotion regulation strategies are also regarded as important antecedents of the experience of shame and guilt. Xu (2024) found that cognitive reappraisal is positively correlated with guilt and negatively correlated with shame, whereas suppression shows a negative correlation with shame and a positive correlation with guilt. At the same time, empirical evidence suggests that some strategies (e.g., suppression) may be more effective in the short term for certain personality profiles, despite their general characterization as being maladaptive (Kobylińska and Kusev, 2019; Kobylińska et al., 2023). Importantly, Shapero et al. (2016) found that cognitive reappraisal moderated the association between temperament-based emotional reactivity and depressive symptoms, suggesting that the psychological consequences of dispositional emotional tendencies may depend on how individuals habitually regulate their emotional responses.

Taken together, a joint consideration of emotional temperament-based dispositions and emotion regulation strategies appears worthwhile for predicting proneness to self-conscious emotions and related outcomes. Because emotional style reflects relatively stable tendencies in emotional reactivity and recovery, whereas emotion regulation strategies reflect habitual ways of managing emotional responses, we examined emotion regulation strategies as potential moderators of the associations between emotional style and shame- and guilt-proneness. This approach was informed by temperament theories that conceptualize both emotional reactivity and self-regulation as components of temperament, with regulatory processes serving to modulate relatively stable emotional tendencies (Rothbart and Bates, 2006; Eisenberg et al., 2010). Importantly, however, these broad temperament-based regulatory capacities are conceptually distinct from the specific, learned emotion regulation strategies (Wright et al., 2025). Research on emotion regulation has shown that individuals differ in their habitual use of specific strategies, such as cognitive reappraisal and expressive suppression, and that these individual differences are associated with psychological and social outcomes (Gross and John, 2003; John and Gross, 2004). Examining such strategies may therefore provide a more specific account of how individuals habitually regulate emotional responses and may help clarify when dispositional emotional tendencies are more or less strongly associated with psychological outcomes. Accordingly, emotion regulation strategies may buffer or amplify the associations between temperament-based emotional dispositions and psychological outcomes. From this perspective, prolonged negative affect following perceived transgressions, reflected in lower positive outlook and slower emotional recovery, may confer greater vulnerability to withdrawal and maladaptive shame-related processes, particularly when emotional experiences are habitually suppressed. Conversely, repeated use of cognitive reappraisal may attenuate such vulnerability by facilitating more adaptive interpretations of emotionally challenging experiences, potentially reducing shame-proneness and supporting more constructive guilt-related responses.

## 4 Methods

### 4.1 Participants

Participants were recruited in Germany via social media and mailing lists and, after giving their informed consent, took part in the survey online via SoSci Survey (Leiner, 2019). The study was approved by the university’s ethics committee prior to data collection. Analyses were carried out using data from a convenience sample of *N* = 331. The respondents were between 16 and 67 years old (*M* = 29.86; SD = 12.00). 273 people were female (82.5%), 52 were male (15.7%) and two were gender diverse (0.6%). Four participants did not provide information on their gender (1.2%). The majority of participants (299; 90.3%) had at least a university entrance qualification. 19 participants (5.7%) had a lower secondary school-leaving certificate or intermediate school-leaving certificate and seven (2.1%) had other educational qualifications; five people (1.5%) did not have a qualification at the time of data collection. One respondent did not provide any information on their educational qualifications (0.3%).

### 4.2 Measures

#### 4.2.1 Emotional style

The dimensions of emotional style were assessed using the Questionnaire of Emotional Style (FEMOS; German: Fragebogen zum emotionalen Stil; Burgstedt et al., 2025). This instrument was developed based on Davidson’s neuroaffective framework (Davidson and Begley, 2012). It is a context-free, economical instrument with a six-dimensional structure (outlook, resilience, self-awareness, social intuition, sensitivity to context, attention). Each scale consists of 4 items, which are to be answered on a scale from 1 (disagree completely) to 7 (agree completely). The Outlook and Resilience scales were used in the present study. In an initial validation study (Burgstedt et al., 2025), the instrument showed acceptable to excellent internal consistencies (*α* = 0.70–0.92), with Outlook (*α* = 0.92) and Resilience (*α* = 0.88) demonstrating excellent and good reliability, respectively.

#### 4.2.2 Shame- and guilt-proneness

A shortened version of the Test of Self-Conscious Affect - Special Populations (TOSCA-SP; Burkert et al., 2017), a further development of the Test of Self-Conscious Affect (TOSCA; Tangney et al., 1989; German translation: Kocherscheidt et al., 2002), was used to assess self-conscious emotions. The TOSCA scales are considered the gold standard for measuring proneness to shame and guilt (Ferguson et al., 2007). They reflect the self-reported individual likelihood of certain evaluations, feelings, and behaviors in situations where social expectations are not met. The abbreviated and adapted version consists of 10 easy-to-understand scenarios describing situations that may occur across diverse lived realities and are not restricted to a specific context. For each scenario, four different possible responses are given, which are rated in terms of their likelihood of occurrence using a 5-point response scale from 1 (unlikely) to 5 (very likely). The four possible reactions are examples of the individual tendency to feel shame or guilt, or the individual tendency to distance oneself or externalize. The present study included only the shame and guilt scales. In a validation study (Burkert et al., 2017), the factors of shame and guilt were confirmed and the adapted TOSCA-SP scales demonstrated good internal consistencies of *α* = 0.89 (Shame) and *α* = 0.81 (Guilt). Although the TOSCA scales are the most widely used measure of shame- and guilt-proneness, several authors have argued that it emphasizes global self-condemnation in shame and reparative responsibility-taking in guilt, thereby capturing predominantly maladaptive aspects of shame and relatively adaptive aspects of guilt rather than the full phenomenology of both emotions (Kubany and Watson, 2003; Tilghman-Osborne et al., 2010; Carnì et al., 2013).

#### 4.2.3 Emotion regulation strategies

A German version of the Emotion Regulation Questionnaire (ERQ; Abler and Kessler, 2009) was employed. It allows for the assessment of habitual preferences for two commonly used emotion regulation strategies, cognitive reappraisal and suppression. The development of the questionnaire was based on Gross’ (1998, 2015) process model of emotion regulation. The scale Cognitive Reappraisal consists of 6 items and Suppression of 4 items, The response scale ranges from 1 to (does not apply at all) to 7 (fully applies). In the validation study (Abler and Kessler, 2009), the two scales demonstrated acceptable internal consistencies of *α* = 0.74 (Cognitive Reappraisal) and *α* = 0.76 (Suppression).

Table 1 reports sample items, means, standard deviations, and internal consistencies for all instruments based on the present sample.

**Table 1:** Measurement instruments, mean values (*M*), standard deviation (SD), internal consistency (*α*).

Scale	Sample item	Number of items	*M*	SD	*α*
*FEMOS* ^1^					
Outlook	My mood is usually bad.*	4	4.84	1.53	0.91
Resilience	Even if something bad happens, I get back on my feet quickly.	4	4.19	1.32	0.85
*ERQ* ^1^		10			
Cognitive Reappraisal	I control my emotions by changing the way I think about the situation I’m in.	6	4.38	1.22	0.88
Suppression	I keep my emotions to myself.	4	3.74	1.32	0.75
*TOSCA-SP* ^2^	While out with a group of friends, you make fun of a friend who is not there.	10			
Shame	You’d feel small and miserable.	10	3.13	0.80	0.80
Guilt	You’d change the subject and talk about the friend’s good points.	10	4.30	0.49	0.71

FEMOS = Questionnaire of Emotional Style; ERQ = Emotion Regulation Questionnaire; TOSCA-SP = Test of Self-Conscious Affect - Special Populations; ^1^response scale 1–7; ^2^response scale 1–5; *item has been inverted.

### 4.3 Data analysis

#### 4.3.1 Preliminary analyses

Analysis was performed using IBM SPSS Statistics 29. Mean values (*M*), standard deviations (*SD*), and Cronbach’s α as a measure of internal consistency were calculated (Table 1). Correlation analyses were carried out to determine the correlations between all constructs and measures used in this study and between age and the other variables (Table 2). Two-tailed *t*-tests were used to identify possible gender effects. The strength of the correlations was measured using Pearson correlations.

**Table 2:** Pearson’s product moment correlations all variables, and gender differences (as yielded by *T*-test).

FEMOS	1	2	3	4	5	6	7
*FEMOS*							
OL (1)	1						
RE (2)	0.62**	1					
*ERQ*							
CR (3)	0.39**	0.39**	1				
ES (4)	−0.14**	−0.08	0.08	1			
*TOSCA* ^2^							
SH (5)	−0.36**	−0.37**	−0.13*	0.26**	1		
GU (6)	0.10	0.04	0.21**	0.05	0.28**	1	
Age (7)	0.12*	0.05	−0.06	−0.01	−0.29**	0.02	1
Mean ± SD							
♀ (*n* = 273)	4.78 ± 1.55	4.09 ± 1.33	4.38 ± 1.26	3.71 ± 1.33	3.20 ± 0.79	4.35 ± 0.46	29.53 ± 11.75
♂ (*n* = 52)	5.32 ± 1.26	4.70 ± 1.14	4.49 ± 0.90	3.80 ± 1.18	2.82 ± 0.74	4.12 ± 0.53	32.15 ± 13.42
*t_gender_* (323) (*d*)	−2.36* (−0.36)	−3.06** (−0.46)	−0.64	−0.46	3.22** (0.49)	3.19** (0.48)	−1.44

FEMOS = Questionnaire of Emotional Style; ERQ = Emotion Regulation Questionnaire; TOSCA-SP = Test of Self-Conscious Affect - Special Populations; OL = Outlook; RE = Resilience; CR = Cognitive Reappraisal; ES = (Expressive) Suppression; SH = Shame; GU = Guilt; *p ≤ 0.05 (two-tailed); **p ≤ 0.01 (two-tailed); *d*, Cohen’s *d*.

#### 4.3.2 Structural path modeling

Structural path modeling was conducted using R with the lavaan package (R Core Team, 2025). Given the focus on testing theoretically derived relationships between constructs rather than the measurement structure itself, the analyses were conducted using a structural path model based on manifest scale scores. This approach also helped to maintain model parsimony and stability, particularly given the inclusion of multiple interaction terms, which would have substantially increased complexity in a full latent variable structural equation model.

**Figure 1:**
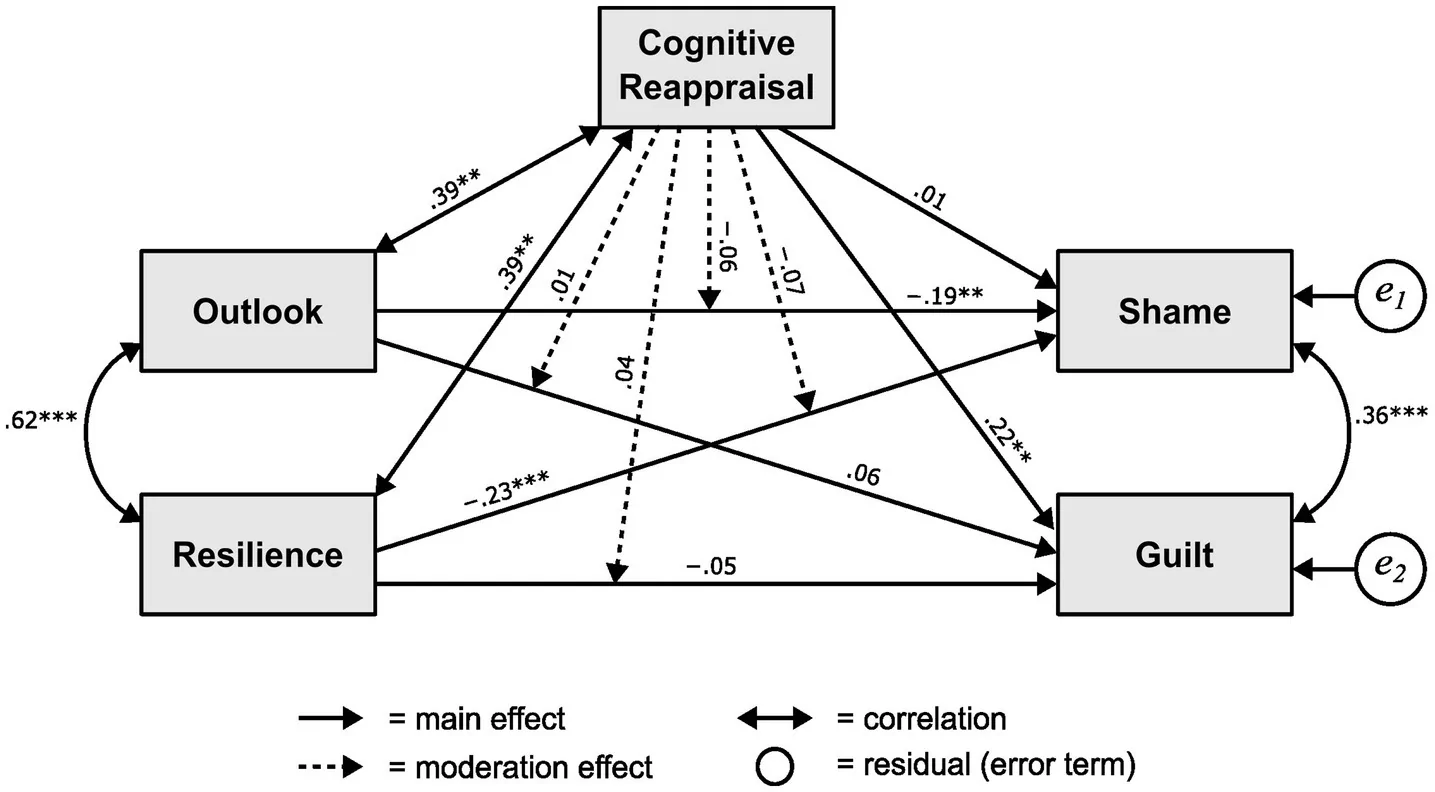
Structural path model predicting shame and guilt with cognitive reappraisal as the moderator. All variables are observed (manifest). Age and gender are not included in this figure for clarity. *p ≤ 0.05, **p ≤ 0.01, ***p ≤ 0.001. This figure was created in Inkscape (Inkscape Project, 2026).

**Figure 2:**
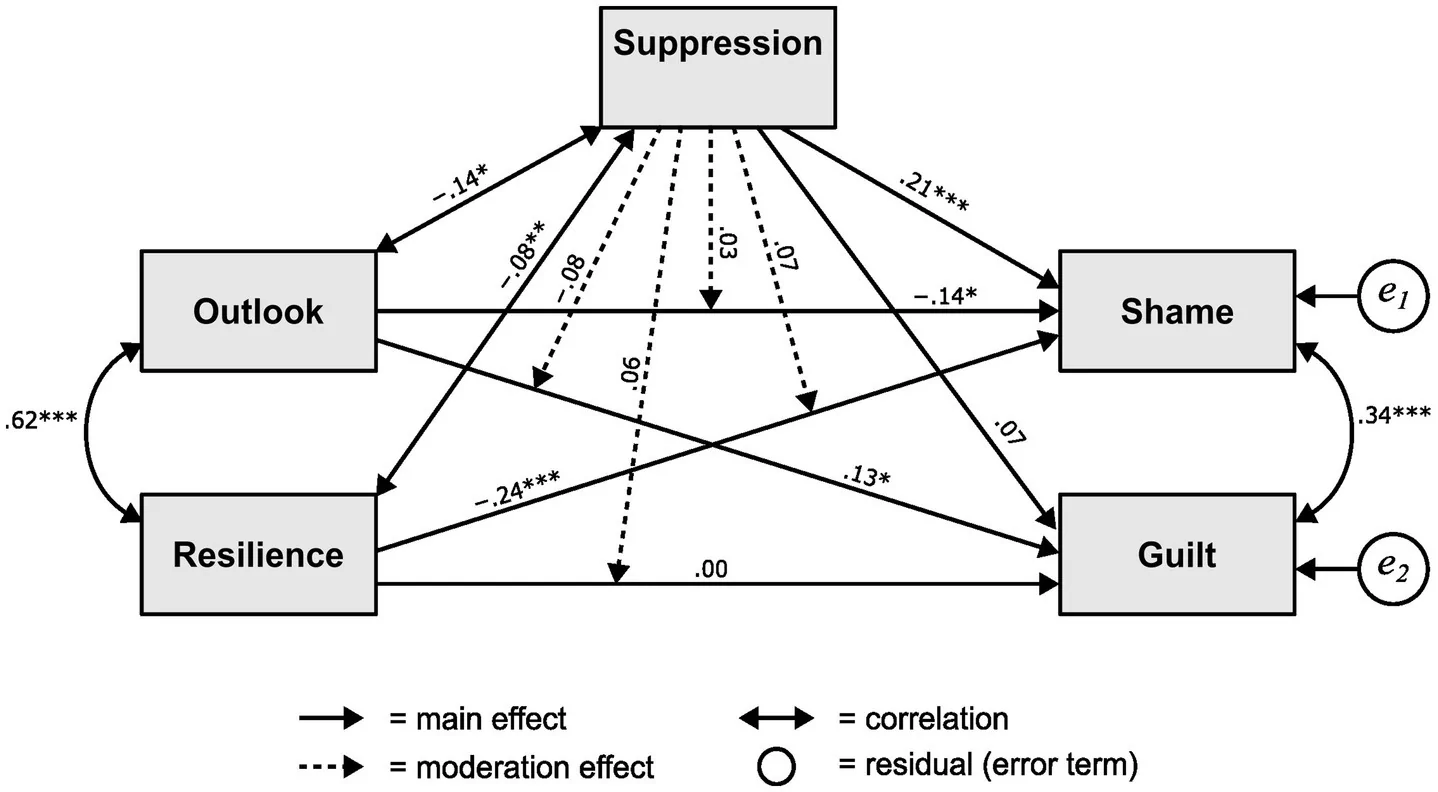
Structural path model predicting shame and guilt with suppression as the moderator. All variables are observed (manifest). Age and gender are not included in this figure for clarity. *p ≤ 0.05, **p ≤ 0.01, ***p ≤ 0.001. This figure was created in Inkscape (Inkscape Project, 2026).

Structural path models were estimated to examine the main effects of outlook, resilience, and emotion regulation strategies (cognitive reappraisal vs. suppression) on shame- and guilt-proneness, as well as interaction effects between emotional style dimensions and regulation strategies. The moderating effects of cognitive reappraisal (Figure 1) and suppression (Figure 2) were also tested in distinct models to reduce complexity and facilitate interpretation of their specific regulatory mechanisms.

Prior to analyses, all continuous predictors and covariates were mean-centered to reduce multicollinearity and to facilitate interpretation of main effects. In each model, interaction terms were computed between the predictor variables (outlook and resilience) and the respective emotion regulation strategy (cognitive reappraisal or suppression). All exogenous predictor variables were allowed to correlate freely, and correlations between the two outcome variables (shame and guilt) were also estimated to account for their shared variance.

To control for potential effects of gender and age, both variables were included as covariates in the structural equation model. Gender was dummy-coded (0 = male, 1 = female) and specified as a direct predictor of the outcome variable, while age was included as a continuous covariate with a direct path to the outcome variable. In addition, both covariates were allowed to correlate with the exogenous predictor variables. Missing data occurred because of participants not providing information on gender or age, as well as two participants who selected the diverse response option for gender. Given the very small number of these cases, they were treated as missing for the purpose of the analyses to maintain model comparability and estimation stability. Missing data were handled using full information maximum likelihood (FIML), as implemented in the model estimation procedure.

Inspection of skewness and kurtosis revealed moderate deviations from normality for several study variables, with some values exceeding commonly used thresholds (|skewness| < 2; kurtosis < 7). Given these deviations, all models were estimated using robust maximum likelihood estimation (MLR), which provides robust standard errors and scaled test statistics.

Statistical power was estimated using Monte Carlo simulations (1,000 replications) based on the sample size (*N* = 331). Data were generated according to the hypothesized multiple regression model including three standardized predictors, two interaction terms, and two covariates. Power was evaluated across three effect size scenarios (conservative, moderate, optimistic) for two outcome variables (shame, guilt), with attenuated effect sizes for guilt. For each simulated dataset, the model was estimated and statistical significance of regression coefficients was assessed at *α* = 0.05. Power was defined as the proportion of simulations in which true effects were correctly detected as significant. Power increased with effect size and was higher for main effects than for interaction effects (Supplementary material 1).

## 5 Results

### 5.1 Preliminary analyses: gender and age effects

Preliminary analyses were conducted to determine which demographic variables needed to be statistically controlled in subsequent structural models. These analyses revealed significant demographic effects across our measures (Table 2). Compared to men, women reported significantly lower levels of positive outlook and resilience compared to men, with small to moderate effect sizes (*d* = −0.36 and *d* = −0.46, respectively). Furthermore, women scored significantly higher on shame- and guilt-proneness (*d* = 0.49 and *d* = 0.48, respectively). Age showed a weak positive association with positive outlook (*r* = 0.12) and shame (*r* = −0.29).

### 5.2 Bivariate associations between variables

Pearson product–moment correlations revealed distinct patterns of associations between outlook and resilience and shame- and guilt-proneness (Table 2). Positive outlook and resilience exhibited negative correlations with shame (*r* = −0.36 and −0.37, respectively). In contrast, guilt was not associated with outlook or resilience. Shame was negatively associated with cognitive reappraisal (*r* = −0.13) and positively associated with suppression (*r* = 0.26). The association between guilt and cognitive reappraisal was weakly positive (*r* = 0.21). No association was found between guilt and suppression. Additionally, cognitive reappraisal was positively associated with both positive outlook and resilience (both *r* = 0.39). Suppression showed a small negative association with positive outlook (*r* = −0.14), whereas its association with resilience was not significant.

### 5.3 Cognitive reappraisal as a moderator

To test whether cognitive reappraisal moderates the relationship between emotional style dimensions and self-conscious emotions, a structural path model was estimated (Table 3, Figure 1). The model demonstrated an excellent fit (*χ*^2^(11) = 9.03, *p* = 0.62, CFI = 1.00, RMSEA = 0.00, SRMR = 0.03) and accounted for 25% of the variance in shame (*R*^2^ = 0.25) and 9% of the variance in guilt (*R*^2^ = 0.09).

**Table 3:** Standardized structural path coefficients, significance tests, and intercorrelations among exogenous and outcome variables (Cognitive reappraisal as moderator).

Predictor	Shame (*b*)	SE	Shame (*β*)	*p*	Guilt (*b*)	SE	Guilt (*β*)	*p*
Outlook	−0.098	0.035	−0.187	0.005	0.019	0.019	0.061	0.308
Resilience	−0.139	0.040	−0.229	<0.001	−0.018	0.023	−0.049	0.429
Cognitive reappraisal	0.007	0.039	0.011	0.861	0.087	0.034	0.219	0.009
Outlook × Cognitive reappraisal	−0.022	0.024	−0.055	0.355	0.002	0.016	0.008	0.900
Resilience × Cognitive reappraisal	−0.034	0.032	−0.073	0.285	0.010	0.017	0.035	0.550
Gender	0.235	0.111	0.108	0.035	0.242	0.081	0.183	0.003
Age	−0.016	0.004	−0.239	<0.001	0.002	0.002	0.045	0.384
Outcome correlation (model-implied)	*r* 0.358	*p* <0.001						

Correlations exogenous variables	1	2	3					
Outlook (1)	–							
Resilience (2)	0.619***	–						
Cognitive reappraisal (3)	0.385***	0.389***	–					
Age (4)	0.106	0.042	−0.068					
Gender (5)	−0.117*	−0.163**	−0.033					

*Significant correlation with *p* ≤ 0.05 (two-tailed); **significant correlation with p ≤ 0.01 (two-tailed); ***significant correlation with *p* ≤ 0.001 (two-tailed).

For shame, both positive outlook and resilience emerged as negative predictors (*β* = −0.19 and *β* = −0.23, *p* < 0.01). For guilt, cognitive reappraisal was a significant positive predictor (*β* = 0.22, *p* < 0.01). None of the tested interaction effects reached statistical significance, with all *p*-values exceeding.

### 5.4 Suppression as a moderator

The second model (Table 4, Figure 2) examined the role of suppression. The model demonstrated an excellent fit(*χ*^2^(11) = 9.18, *p* = 0.61, CFI = 1.00, RMSEA = 0.00, SRMR = 0.03)and accounted for 28% of the variance in shame (*R*^2^ = 0.28) and 5% of the variance in guilt (*R*^2^ = 0.05).

**Table 4:** Standardized structural path coefficients, significance tests, and intercorrelations among exogenous and outcome variables (suppression as moderator).

Predictor	Shame (*b*)	SE	Shame (*β*)	*p*	Guilt (*b*)	SE	Guilt (*β*)	*p*
Outlook	−0.075	0.034	−0.144	0.029	0.041	0.020	0.127	0.040
Resilience	−0.145	0.038	−0.239	<0.001	−0.001	0.021	−0.004	0.951
Suppression	0.129	0.030	0.214	<0.001	0.026	0.024	0.070	0.276
Outlook × Suppression	0.012	0.023	0.032	0.608	−0.019	0.015	−0.083	0.217
Resilience × Suppression	0.029	0.030	0.067	0.340	0.016	0.014	0.060	0.250
Gender	0.229	0.106	0.106	0.030	0.254	0.081	0.192	0.002
Age	−0.016	0.003	−0.241	<0.001	0.001	0.002	0.025	0.060
Outcome correlation (model-implied)	*r* 0.343	*p* *<0*.001						

Correlations exogenous variables	1	2	3					
Outlook (1)	–							
Resilience (2)	0.619***	–						
Suppression (3)	−0.137*	−0.076	–					
Age (4)	0.105	0.042	−0.013					
Gender (5)	−0.117*	−0.164**	−0.028					

*Significant correlation with *p* ≤ 0.05 (two-tailed); **significant correlation with *p* ≤ 0.01 (two-tailed); ***significant correlation with *p* ≤ 0.001 (two-tailed).

For shame, both positive outlook and resilience emerged as negative predictors (*β* = −0.14 and *β* = −0.24, *p* < 0.05). Suppression emerged as a positive predictor of shame (*β* = 0.21, *p* < 0.001). Including suppression in the model, positive outlook exhibited a positive main effect on guilt (*β* = 0.13, *p* = 0.04). None of the tested interaction effects reached statistical significance, with all *p*-values exceeding 0.21.

## 6 Discussion

This study investigated the interplay between emotional style, as temperamental dimensions (Burgstedt et al., 2025; Davidson and Begley, 2012), and learned emotion regulation strategies in the regulation of self-conscious emotions. Specifically, the study examined whether the emotional style dimensions outlook and resilience were associated with proneness to shame and guilt and whether these associations varied as a function of the emotion regulation strategies cognitive reappraisal and suppression.

Positive outlook and resilience were negatively associated with shame-proneness, whereas associations with guilt-proneness were not significant. Shame-proneness was also positively associated with suppression and negatively associated with cognitive reappraisal, whereas guilt-proneness was positively related to cognitive reappraisal. These patterns seem consistent with theoretical accounts emphasizing shame as involving more global and stable self-evaluative processes (“I am bad”) (Tangney and Dearing, 2002). In contrast, guilt-proneness (“My action was bad”) may be more strongly linked to appraisal-based regulatory processes. However, the stronger links between shame-proneness and the two emotional style dimensions may partly reflect the greater overlap of the operationalization of shame-proneness with broader affective vulnerability. This interpretation highlights the importance of distinguishing between dispositional measures of shame- and guilt-proneness and shame as a context-dependent moral-relational experience that can vary in its manifestations, functions, and consequences across situations. The observed associations may therefore primarily reflect individual differences in persistent negative self-focused processing and affective regulation, rather than a direct association between emotional style and the broader range of ways in which shame may be experienced and expressed in specific contexts.

We next considered whether the associations of the two emotional style dimensions with shame- and guilt-proneness differed depending on individuals’ habitual use of emotion regulation strategies. All structural path models predicting shame and guilt demonstrated an excellent model fit due to the simple observed-variable structure of the models, since no latent measurement models were specified. Given that the present analyses were based on observed scale scores, all associations reported should be interpreted as reflecting relationships among measured indicators, which may be affected by measurement error.

In the prediction of shame-proneness, both positive outlook and resilience showed consistent negative associations with shame-proneness, irrespective of whether cognitive reappraisal or suppression was included as the emotion regulation strategy. Lower levels of positive affect maintenance and slower recovery from aversive events may therefore represent dispositional vulnerabilities associated with greater shame-proneness. This finding is consistent with the notion that shame-proneness is characterized by persistent negative self-evaluation (Tangney and Dearing, 2002; Tracy and Robins, 2004). One possibility is that lower resilience may be associated with prolonged negative affective responses that, in turn, could contribute to more persistent self-evaluative processes characteristic of shame. Whether such processes are indeed linked to resilience-related differences in neural recovery mechanisms, such as prefrontal regulation of amygdala responses (Davidson and McEwen, 2012; Kim and Whalen, 2009), remains an empirical question for future research. Suppression was positively associated with shame-proneness. This association may reflect that habitual inhibition of emotional expression limits opportunities for emotional processing and interpersonal communication following self-relevant experiences. This may, in turn, be linked to withdrawal-oriented responses characteristic of shame-related processes (Lindsay-Hartz, 1984; Tangney, 1995; Tangney and Dearing, 2002). Rather than specifically amplifying the effects of prolonged negative affect, suppression may represent a broader regulatory tendency associated with shame-proneness across different levels of emotional style. A different pattern emerged for guilt-proneness. Positive outlook was associated with guilt-proneness, but only when suppression was included in the model. Perhaps maintaining positive affect can encourage a constructive engagement with self-relevant failures, enabling individuals to accept responsibility (Baumeister et al., 1994; Tangney, 2002; Tangney and Dearing, 2002). However, this association was no longer evident in the context of cognitive reappraisal. One possible explanation is that positive outlook and cognitive reappraisal reflect related ways of approaching emotionally challenging experiences. Individuals with a more positive outlook may be more likely to engage in positive re-evaluation, i.e., cognitive reappraisal, which could in turn be related to a greater tendency to experience guilt in response to perceived wrongdoing. Although the cross-sectional design does not allow us to determine the direction of these relationships or to establish mediation, this possibility suggests that cognitive reappraisal may represent one process through which positive outlook is linked to guilt-proneness. At the same time, positive outlook and cognitive reappraisal both involve a constructive approach to emotional experiences, making it difficult to disentangle their respective associations with guilt-proneness when they are considered together. In contrast, suppression reflects a different way of managing emotional experiences, namely inhibiting their outward expression. The association between positive outlook and guilt-proneness may therefore be more apparent when suppression is included, as the two constructs capture more distinct aspects of emotional functioning.

Overall, positive outlook was associated with both shame- and guilt-proneness, suggesting that a general tendency to maintain positive affect and a constructive orientation toward future experiences may be relevant for self-conscious emotional responses more broadly. Such an orientation may help individuals acknowledge personal shortcomings without engaging in global negative self-evaluation, while also supporting the perception that mistakes can be repaired. In contrast, resilience was associated with shame-proneness but not guilt-proneness, suggesting that the capacity to recover from aversive emotional states may be particularly relevant for limiting maladaptive shame-related processes but is not necessarily sufficient to foster reparative guilt responses. Suppression was associated with shame-proneness, consistent with the withdrawal-oriented tendencies often associated with shame. In contrast, guilt-proneness, which is characterized by responsibility-taking and reparative action tendencies, may be more closely aligned with the acknowledgment and cognitive processing of one’s role in a negative event. Consistent with this interpretation, cognitive reappraisal, a strategy reflecting the tendency to reinterpret emotionally challenging situations in a more adaptive manner, was associated with guilt-proneness.

Contrary to expectations, we found no evidence that habitual emotion regulation strategies modified the associations between emotional style dimensions and shame- and guilt-proneness in the present sample. At the same time, the associations between emotion regulation strategies, particularly cognitive reappraisal, and both positive outlook and resilience indicate that these constructs share relevant variance. This pattern suggests that emotional style and emotion regulation may represent interconnected aspects of emotional functioning that could contribute to shame- and guilt-proneness through overlapping or sequential processes. Another possibility is that the relationships between emotional style, emotion regulation, and self-conscious emotions may not be fully captured by linear interaction models. For example, regulatory strategies may be differentially related to self-conscious emotions at different levels of emotional style, such that suppression or reappraisal may have distinct implications for individuals with particularly low versus high levels of outlook or resilience. Furthermore, contextual factors such as goal relevance may shape how emotional information is processed and translated into emotional responses. Recent systematic review and meta-analytic evidence suggests that the effects of emotional information depend in part on its relevance to an individual’s current goals (Mirabella and Montalti, 2025; Mirabella et al., 2026). In this context, emotion regulation strategies may moderate the associations between emotional style dimensions and self-conscious emotions particularly when emotional stimuli or events are perceived as relevant to an individual’s current goals. If an emotional event is not relevant to an individual’s current goals, the habitual use of a particular regulation strategy may be less likely to shape how dispositional emotional tendencies relate to subsequent emotional responses. Goal relevance may therefore represent an additional contextual factor that could help explain why no significant moderation effects were observed in the present study.

Given the relevance of shame- and guilt-related processes to social and antisocial behavior, particularly in the context of prevention, intervention, and rehabilitation (Ewald, 2018; Hosser et al., 2008; Spruit et al., 2016; Tangney et al., 2007a, 2011a, 2011b, 2014; Tibbetts, 2003), the present findings provide valuable insights. Within Davidson’s framework, interventions targeting outlook would focus primarily on enhancing and sustaining positive emotional states (Davidson and Begley, 2012). Practices such as savoring positive experiences, gratitude exercises, compassion-based meditation, and the cultivation of positive social connections may therefore be particularly relevant for strengthening positive outlook, as they are thought to promote the maintenance of positive affect over time. Mindfulness-based approaches may support resilience-related recovery processes by promoting more adaptive responses to emotionally challenging experiences. A positive outlook and greater emotional resilience may be associated with more adaptive self-conscious emotional responses. However, whether these are linked to more prosocial forms of guilt and reduced vulnerability to shame-related processes, such as “shame-rage” cycles associated with hostility and aggression (Hosser et al., 2008; Spruit et al., 2016), remains an empirical question. At the same time, effective interventions should also promote adaptive emotional regulation strategies such as cognitive reappraisal, which can be improved through structured interventions involving cognitive restructuring, reinterpretation, and perspective shifting (Gross, 1998; Denny and Ochsner, 2014).

Nevertheless, several limitations of the present study should be acknowledged. The cross-sectional design precludes causal inferences, so further longitudinal research is needed to determine how emotional style and habitual emotion regulation strategies influence one another over time (Burgstedt et al., 2025; Kim and Whalen, 2009). Future research should also examine whether nonlinear or person-centered approaches provide additional insights into these complex relationships. Online data collection may have limited the study’s reach, potentially excluding certain participant groups. The present sample consisted predominantly of German, highly educated participants and therefore reflects self-conscious emotional processes within a Western cultural context. Cultural norms influence both the experience of shame and guilt (Silfver-Kuhalampi et al., 2013) and the perceived adaptiveness of emotion regulation strategies. In particular, although suppression has frequently been associated with less adaptive psychological outcomes in Western samples (Gross and John, 2003), accumulating evidence suggests that these associations are not culturally universal. In more collectivistic cultural contexts, where emotional restraint is more strongly normatively valued, suppression has been shown to be less consistently associated with impaired psychological functioning and adverse interpersonal consequences (Butler et al., 2007; Soto et al., 2011). Accordingly, the present findings should be interpreted within the cultural context in which the data were collected and require replication in more culturally diverse samples. Additionally, although our sample size (*N* = 331) provides sufficient power, future studies should replicate these patterns in more representative samples, as well as in clinical and forensic populations, where shame and guilt processes are often more deeply pathological (Ewald, 2018; Tibbetts, 2003). Furthermore, although gender was statistically controlled in the present analyses, we cannot exclude the possibility that effects may vary across gender groups. Given the substantial gender imbalance in our sample, gender-specific invariance of the moderation effects could not be reliably examined. Future research with more balanced samples is needed to determine whether the observed associations between emotional style, emotion regulation, and shame- and guilt-proneness generalize across genders. Further studies should also examine the role of situational factors (and their goal relevance) in shaping the experience of shame and guilt, as the phenomenology of these self-conscious emotions likely emerges from the interplay between stable individual differences and contextual variability. An additional consideration concerns the modeling approach. As the present analyses did not include full latent variable structural equation models, measurement error was not explicitly accounted for. This may have influenced the observed associations. Moreover, experimental designs may further advance this line of research by enabling a more controlled investigation of the elicitation and regulation of shame and guilt.

Taken together, the present findings support a complementary, rather than a competing, view of emotional style (Davidson and Begley, 2012; Kesebir et al., 2019) and habitual preferences of emotion regulation strategies (Abler and Kessler, 2009; Gross, 2015; Gross and John, 2003). Emotional style can be conceptualized as a temperamental blueprint that reflects baseline tendencies in emotional responses. In contrast, emotion regulation strategies are experience-dependent components that also contribute to emotional outcomes. This pattern underscores the importance of integrating trait- and process-oriented perspectives to fully capture the emergence and modulation of self-conscious emotions. Both frameworks may be promising targets for emotion-focused interventions, as they operate at different but interrelated levels of emotional functioning.

## Data Availability

The raw data supporting the conclusions of this article will be made available by the authors, without undue reservation.
