# Supplementary material for: When style meets strategy: testing the moderating role of regulation strategies in the association between emotional style and self-conscious emotions
Source: Front Psychol. 2026 Sep 9;17:1913847. doi: 10.3389/fpsyg.2026.1913847 (PMC13597371; doi:10.3389/fpsyg.2026.1913847)
Supplement: Supplementary file 1 [file Table_1.DOCX]

Supplementary Material

# 1 Power Analysis using Monte Carlo Simulations

**Table 1.** Statistical Power based on Monte Carlo simulations (1,000 replications, *N* = 331)

| **Effect type** | **Scenario** | **Shame power** | **Guilt power** |
| --- | --- | --- | --- |
| Main effects | Conservative | 0.726-0.926 | 0.441-0.706 |
|  | Moderate | 0.990-0.998 | 0.912-0.992 |
|  | Optimistic | 0.997-1.000 | 0.987-1.000 |
| Interaction effects | Conservative | 0.403-0.440 | 0.215-0.238 |
|  | Moderate | 0.734-0.759 | 0.544-0.558 |
|  | Optimistic | 0.915-.0.933 | 0.844-0.850 |

*Note.* Values represent the proportion of simulations in which the tested effect was significant at α = .05.
